# Dynamics of the Microbiome and Antibiotic Resistome in Hyper-Mesophilic Anaerobic Digestion of Cattle Manure Assisted with Granular Activated Carbon

**DOI:** 10.1021/acsenvironau.5c00239

**Published:** 2026-02-18

**Authors:** Mac-Anthony Nnorom, Bang Du, Zhufang Wang, Zilin Tian, Rupert Hough, Lisa Avery, Devendra Saroj, Bing Guo

**Affiliations:** † School of Engineering, 3660 University of Surrey, Guildford GU2 7XH, U.K.; ‡ The James Hutton Institute, Craigiebuckler, Aberdeen AB15 8QH, U.K.

**Keywords:** antimicrobial resistance, granular activated carbon, anaerobic digestion, cattle manure, hyper-mesophilic temperatures

## Abstract

The use of conductive materials, such as granular activated carbon (GAC), for optimization of the anaerobic digestion (AD) process has garnered attention in recent years; however, its impact on the dynamics of the microbiome and resistome in continuous AD systems remains unclear, especially under temperature variation. This study combined culture-based bacterial enumeration and shotgun metagenomics to investigate the impact of two GAC application strategies, suspended and packed, on the fate of pathogens (viable *Escherichia coli*) and ARGs during the AD of cattle manure at 40 and 45 °C. The results show that GAC mitigated the process imbalance and shock induced by temperature transition. The microbial community in the AD sludge was highly impacted by temperature but not GAC, while GAC biofilms showed notably higher archaeal abundance. All AD reactors reduced viable *E. coli*, with the highest reduction occurring in the packed GAC reactors (95.70–96.24%), followed by the suspended GAC (94.53–95.69%), and then the non-GAC (92.77–94.24%). Culturable tetracycline-resistant bacteria were reduced below the quantification limit in all reactors. Reduction of ampicillin-resistant bacteria showed stochastic trends at 40 °C but improved at 45 °C, indicating limited impact by GAC. ARGs and mobile genetic elements (MGEs) were reduced in all reactors at comparable levels, regardless of GAC addition. Temperature transition exerted a mixed effect, with higher reduction of some resistance classes (MLS, tetracycline, and multidrug) and lower reduction of others (bacitracin, aminoglycoside, beta-lactam, and streptothricin). Mantel test and Procrustes analysis revealed a significant correlation between the resistome and the bacterial community, inferring that shifts in the ARG host population were a major determinant of the fate of ARGs. Overall, GAC was beneficial to reactor stability but had a minimal influence on the reduction of *E. coli*, ARGs, and MGEs. It is highly recommended to monitor antimicrobial resistance using both culture-based and culture-independent methods.

## Graphical abstract

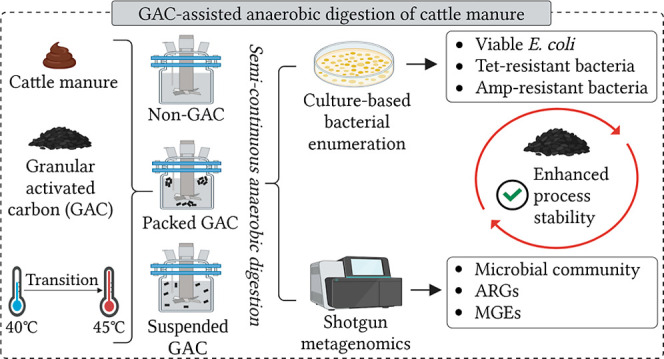

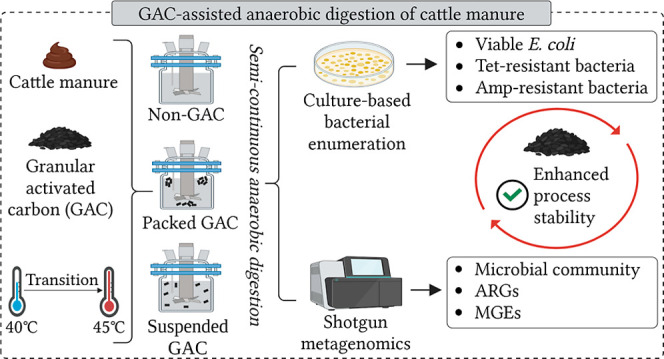

## 1 Introduction

The evolution and propagation of antimicrobial resistance (AMR) in agroecosystems is a contemporary challenge with profound implications for global health. Beyond clinical settings, antibiotics are extensively used in animal husbandry for the treatment and control of infectious diseases. Therapeutic and prophylactic administration of antibiotics to livestock can impose strong selection pressure on the gut microbiota. This, in turn, fosters the proliferation of antibiotic-resistant bacteria (ARB) as well as the mobilization and horizontal transfer of antibiotic resistance genes (ARGs).^1,2^ Consequently, livestock manure often harbors antibiotic residues and diverse bacterial populations that possess intrinsic and acquired resistance against several frontline antibiotics used in human and veterinary medicine.^3,4^ Given the burden of resistance determinants in livestock manure, it is crucial to implement robust management measures to mitigate the risk of AMR transmission to humans, animals, and the environment.

Anaerobic digestion (AD) is a sustainable approach widely adopted for the management of organic wastes. It is leveraged in the agricultural sector to stabilize and disinfect livestock manure while recovering renewable energy in the form of biogas. The digestate is an ideal soil amendment and fertilizer; however, there is a growing concern that, akin to untreated manure, its use on farmland could represent a route for the dissemination of AMR to the food chain and water resources.^5^ This concern stems from the increasing recognition that ARB, ARGs, and antibiotic residues are seldom completely removed through AD and may persist in the digestate depending on the reactor configuration and operating conditions, such as temperature and retention time.^6,7^ For example, Sun et al.^8^ and Flores-Orozco et al.^9^ reported the enrichment of fluoroquinolone, tetracycline, and sulfonamide resistance genes during the AD of cattle manure at 35 °C, irrespective of antibiotic spiking. In a similar vein, the survey of full-scale agricultural biogas sites in the UK revealed that, despite a substantial reduction in ARG levels (41–92%), the digestate from manure-fed digesters harbored remnants of high-risk ARGs linked to human and animal pathogens.^10^ Although the risk of human colonisation and infection via the food chain is still poorly defined, recent studies have shown that the application of digestate to land can enrich the soil resistome and result in increased contamination of fresh produce by ARB.^5,11,12^ From this perspective, greater effort needs to be devoted to the optimization of AD to enhance the attenuation of pathogens and ARGs.

The application of carbon-based conductive materials, such as granular activated carbon (GAC) and biochar, to manure AD has emerged as a practical, relatively low-cost optimization option capable of improving operational efficiency, stability, and performance.^13^ These materials are functionally versatile and have been demonstrated to possess remarkable properties that make them well-suited and beneficial to AD. For instance, GAC exhibits high electrical conductivity, and more importantly, its inherent porosity provides a large surface area for microbial attachment and contaminant adsorption.^14^ As a prime AD additive, GAC can promote direct interspecies electron transfer (DIET) between functional microbial guilds, leading to accelerated substrate degradation and higher methane output.^15,16^ In addition to methane recovery, the capacity of GAC to influence the fate of ARGs has generated interest in recent times, with the few existing studies reporting contradictory findings.^17,18^ On the one hand, GAC was revealed to facilitate a reduction in the abundance of some sulfonamide, tetracycline, macrolide, and beta-lactam resistance genes under oxidative stress from nanoplastics.^17^ Conversely, under ammonia stress, the enrichment of intracellular ARGs and the attenuation of extracellular ARGs were observed.^18^ While the role of GAC in AD is broadly underpinned by its physicochemical properties, it remains unclear whether the dynamics of ARGs and bacterial pathogens in GAC-assisted AD are indeed selective (reproducible) or predominantly stochastic. Furthermore, in relation to AMR, very little is known about the long-term performance of GAC in continuously operated digesters. Previous studies using batch tests may not represent the true effects of GAC on AMR. This is an important knowledge gap given that the continuous mode of operation more closely reflects real-world practice and could contribute to divergence in the microbial community and antibiotic resistome over time.

For the successful deployment and upscaling of GAC-assisted AD, a methodical approach to GAC application or dosing is paramount, particularly in conventional systems like continuous stirred-tank reactors (CSTRs). In most use cases, the maintenance of GAC in suspension is preferable as sedimentation could potentially limit contact between cells and GAC, thereby affecting efficacy.^19^ Nonetheless, GAC fluidization is caveated by the increase in the loss of GAC upon digestate withdrawal. This limitation has a bearing on the operational costs because of the need for frequent dosing to compensate for washout. In addition, the accumulation of GAC in the digestate could trigger quality compliance concerns, complicating its downstream use and management. A viable alternative is to load GAC on mobile carriers so that it not only remains in suspension but can also be easily retrieved from the digestate and recycled.^13^ Despite the perceived prospects, the impact of this approach on the functionality of GAC is still poorly understood.

The present study aims to systematically investigate the impact of suspended (fluidized) and packed GAC (embedded in mesh carriers) on the microbiome and antibiotic resistome in cattle manure anaerobic digesters operated semicontinuously under hyper-mesophilic temperatures (40 and 45 °C). Culture-based bacterial enumeration was combined with shotgun metagenomics to (a) uncover taxonomic and functional diversity, (b) track microbial community succession, (c) monitor pathogen removal, and (d) determine the abundance, potential hosts, and mobility of ARGs. This study takes a deep dive into the AMR mitigation prospects of GAC-assisted AD systems, bridging gaps and generating valuable insights necessary for empirical design and adoption at scale.

## 2 Methodology

### 2.1 Reactor Setup and Operation

A bioreactor simulator unit was used for the AD of cattle manure (Bioprocess Control, Sweden). The unit comprises six CSTRs marked R1 to R6. Two of the reactors, R1 and R2, were replicate controls without GAC. They were designated as “non-GAC”, each containing two empty mesh media bags (rated 450 μm, Aquatic Experts, USA). R3 and R4, on the other hand, were designated as “packed GAC” and each contained mesh media bags filled with GAC (4–12 mesh, Merck, UK) to a total concentration of 5 g/L (relative to the reactor working volume). R5 and R6 also received 5 g/L of GAC, but it was added directly without the use of mesh bags. These two reactors were designated as “suspended GAC”. Before addition, GAC was soaked overnight in deionized water and rinsed repeatedly to remove impurities. The physical properties of the GAC used in this study can be found in Wang et al.^20^

All six reactors had a working volume of 1.7 L and were operated in parallel under semicontinuous feeding mode for over 250 days. For the duration of reactor operation, the organic loading rate (OLR) was maintained at 2.6 g VS L^–1^ d^–1^, while the solids retention time (SRT) was maintained at 20 days. A 20 day SRT was adopted with the intent to ensure a good balance between biomass retention, process stability, and performance. This is consistent with previous studies of manure-based AD, which have demonstrated stable methanogenesis under SRTs of 15 days and above.^21–23^ Given the use of CSTRs with no separation or retention of solids, the SRT was assumed to be equal to the hydraulic retention time (HRT) and therefore calculated as the ratio of the working volume (L) and flow rate (L^–1^·d^–1^). Feeding and discharge occurred every 3 days, with the withdrawal of 255 mL of digestate and the addition of an equivalent volume of manure. This three-day interval was implemented for practical reasons. Before feeding, the manure was diluted with deionized water to achieve 5.1% volatile solids (VS). The headspace of the reactors was flushed with nitrogen gas after feeding to maintain anaerobic conditions. Each reactor was equipped with a mechanical stirrer running at a speed of 120 rpm. Mixing occurred intermittently, once every hour for 10 min, with the stirrer alternating between clockwise and anticlockwise rotation at 15 s intervals. An online gas measuring device automatically logged the biogas flow rate. The experimental period was categorized into two distinct phases based on the operating temperature of the reactors: phase I (40 °C, days 126–206) and phase II (45 °C, days 207–249). Phase I commenced following a switch of feedstock from freshly voided cattle faeces to the solid fraction of mechanically separated cattle manure. The latter was obtained in bulk from a full-scale AD plant in the UK and stored at −20 °C until use. Its characteristics are presented in Table S1 (Supporting Information 1). To minimize acid inhibition during phase II, pH was adjusted and kept within 6.8–7.1 through the addition of 1 M NaOH.

Routine physicochemical analysis, including total solids (TS), volatile solids (VS), chemical oxygen demand (COD), and ammoniacal nitrogen, was measured according to standard methods.^24^ Biogas composition was analyzed using a gas chromatograph equipped with a thermal conductivity detector (Agilent Technologies, USA). The Fourier Transform Infrared (FTIR) gas analyzer (Gasmet Technologies, Finland) was also used for biogas composition testing. The concentration of volatile fatty acids (VFAs) was quantified using a gas chromatograph equipped with a flame ionization detector (Agilent Technologies, USA).

### 2.2 Culture-based Bacterial Enumeration

The prevalence of *Escherichia coli* (used here as an indicator pathogen) and culturable ARB in the raw manure and digestate samples were assessed via agar plating. First, a 10-fold primary dilution was prepared by adding 90 mL of sterile Ringer’s solution to 10 g of manure or digestate. The suspension was homogenized by thorough agitation on a roller mixer for 1 h at a speed of 200 rpm. Further dilutions were prepared from the primary dilution and subsequently filtered under vacuum through 0.45 μm membrane filters. The filtration step was performed in duplicate, and each filter was aseptically transferred to an agar plate containing membrane lactose glucuronide agar (MLGA). The plates were incubated initially at 30 °C for 4 h and then at 44 °C for 18 h. Following incubation, green and blue colonies on the filters were counted and recorded as *E. coli*. Colony counting was limited to plates with 15 to 80 discrete colonies (quantification limit).

To enumerate presumptive ARB, 100 μL of the primary and secondary dilutions were directly spread, in duplicate, on the surface of Brain Heart Infusion (BHI) agar containing either ampicillin (40 μg/mL) or tetracycline (25 μg/mL). Both antibiotics were selected based on clinical relevance and widespread administration to food-producing animals in the UK.^25^ The concentration of the antibiotics was predicated on published breakpoints.^26,27^ After incubation at 37 °C for 24 h, colony counts were recorded for plates with colonies ranging between 30 and 300.

### 2.3 DNA Extraction and Shotgun Metagenomic Sequencing

Samples were collected from the six reactors at the start and end of each experimental phase for molecular analysis. This consisted of one raw manure sample, 24 digestate samples, and two GAC samples (Table 1). The GAC samples were obtained from the packed GAC reactors at the end of phase II and gently rinsed thrice with deionized water to remove loosely bound microbial biomass. Genomic DNA was extracted from the samples using the NucleoSpin Soil kit (Macherey-Nagel, Germany), in accordance with the manufacturer’s protocol. DNA extracts were shipped to the Centre for Genomic Research, Liverpool, for library preparation and paired-end metagenomic sequencing (2 × 150 bp) on the Illumina NovaSeq platform. Quality control of the raw sequence data was performed with cutadapt v4.5 to remove low-quality reads (<20 q-score) and adapters.^28^ The command line options were set as follows: -O 3, -q 20, -m 15. An average of 103 million clean reads was generated per sample after trimming and filtering.

**Table 1:** Details of Samples Subjected to Shotgun Metagenomics

sample type	phase^a^	temperature (degC)	description	reactors sampled^b^	number of samples	sampling points
raw manure	–	–	solid fraction of mechanically separated cattle manure	–	1	–
digestate	phase I-SP	40	stable (start of phase I)	R1–R6	6	day 147
	phase I-EP		stable (end of phase I)	R1–R6	6	day 195
	phase II-SP	45	transition (start of phase II)	R1–R6	6	day 222
	phase II-EP		recovery and adaptation (end of phase II)	R1–R6	6	day 249
GAC samples	phase II-EP		recovery and adaptation (end of phase II)	R3 and R4	2	day 249

a SP stands for start point, whereas EP stands for end point.

b R1 & R2: non-GAC reactors; R3 & R4: packed GAC reactors; R5 & R6: suspended GAC reactors.

### 2.4 Read-Based Taxonomic Profiling

Taxonomy was assigned to the clean reads using Kraken 2 v2.1.2 and the standard reference database (released 02–04–2025).^29^ Bracken v3.0.1 was used to estimate taxa abundance at the species and genus levels.^30^ As noted in Nnorom et al.,^10^ the rationale for selecting Kraken 2 and Bracken for microbial community characterization was driven by processing speed, precision, and computational resource efficiency.

### 2.5 Annotation of ARGs and Mobile Genetic Elements (MGEs)

The abundance of ARGs and MGEs was computed by using ARGs-OAP v3.2.4 with default settings (--*e* ≤ 1 × 10^–7^, --id ≥ 80, --length ≥ 25) to align the clean reads against the structured ARG (SARG) database and a curated MGE database.^31,32^ Contig-based annotation of ARGs and MGEs followed a previously described workflow.^10^ In brief, clean reads were coassembled with Megahit v1.2.9 and then mapped back to the assembly with BWA-MEM.^33,34^ Contigs greater than 500 bp in length were retained in the assembly and screened for ARGs and MGEs using ABRicate v1.0.1 in conjunction with the SARG and MGE databases.^35^ A threshold value of ≥80% was applied for gene identity and coverage. ARG and MGE-bearing contigs were classified at the species level with Kraken 2 v2.1.2.^29^

### 2.6 Statistical Analysis

Principal coordinate analysis (PCoA), permutational multivariate analysis of variance (PERMANOVA), and permutational analysis of multivariate dispersions (PERMDISP) were conducted based on Bray–Curtis dissimilarity to explore compositional differences in the microbiome and resistome of samples and groups. The correlation between the microbial community, ARGs, and MGEs was evaluated with the Mantel test and Procrustes analysis. Intraclass correlation coefficient (ICC) and one-way Analysis of Variance (ANOVA) were used to check the variability of analytical measurements. Differential abundance analysis of ARGs was performed with DESeq2.^36^ Outcomes of statistical tests were deemed significant if the p-value fell below 0.05.

## 3 Results and Discussion

### 3.1 Changes in Reactor Performance during Long-Term Operation

The biogas productivity of the non-GAC (R1 and R2), packed GAC (R3 and R4), and suspended GAC (R5 and R6) experimental groups are depicted in Figure S1 (Supporting Information 1). In the start-up period leading up to phase I (days 1–86), freshly voided cattle dung served as feedstock, and the steady-state biogas production was consistent between replicate reactors (ICC = 0.70–0.93, *p* < 0.05) and across the three groups (ANOVA, *p* > 0.05). A switch of feedstock to separated cattle manure was initiated on day 87 and followed by a transition period of 40 days (2 SRTs) to allow for microbial acclimation. The reactors reached a new steady state after day 123, marking the onset of phase I. During this phase, the performance of replicate reactors became less consistent as indicated by the ICC obtained for non-GAC (0.74, *p* < 0.05), packed GAC (0.48, *p* < 0.05), and suspended GAC (0.62, *p* < 0.05). The increase in the variability of replicates may have stemmed from the heterogeneity of the separated cattle manure, which had a range of constituents, including faeces, bedding, and bits of plant residues. Nonetheless, the average daily biogas production, pH (6.8–7), and VS removal (34.06–36.88%) remained comparable across the three experimental groups.

Phase II commenced on day 207 following a one-step increase in the operating temperature from 40 to 45 °C. The abrupt temperature change triggered an immediate decline in biogas production and VS removal (28.86–30.82%). This effect was more profound in non-GAC reactors and could be attributed to VFA accumulation and the inhibition of sensitive microbial populations.^37,38^ By contrast, packed GAC reactors exhibited greater stability and resilience to temperature shock. They recovered faster with minor variability between them (ICC = 0.82, *p* < 0.05).

Collectively, the results revealed that although GAC did not have an obvious impact on the biogas output during long-term operation, its benefit to manure AD was reflected in the enhanced capacity to tolerate process disturbance and shock induced by temperature change. A similar finding was previously reported under stress from substrate overloading.^39,40^ Both studies found that reactors amended with GAC and powdered activated carbon (PAC) outperformed the control solely when the OLR was increased to very high levels. The stress mitigation function of GAC may stem from a combination of inherent properties, including the buffering of pH, the adsorption of toxic intermediates (e.g., ammonia), and the retention of biomass.^41,42^

### 3.2 Microbial Community Diversity, Composition, and Succession

#### 3.2.1 Bacterial Population

The PCoA ordination plot revealed that temperature strongly influenced the bacterial community structure (Figure 1A). As denoted by the spatial distance between phases, the community significantly diverged over time in response to temperature change (PERMANOVA: adonis2, R^2^ = 0.82, F = 35.64, p = 0.0001; PERMDISP: p = 0.19), while the non-GAC, packed GAC, and suspended GAC reactors closely clustered at each phase, suggesting that the impact of GAC on the community structure was not significant (PERMANOVA: adonis2, R^2^ = 0.04, F = 2.57, p = 0.048; PERMDISP: p = 0.89). Regardless, the GAC biofilm samples obtained from R3 and R4 were distant from all sludge samples but overlapped with each other, indicating high reproducibility and colonisation of the GAC surface by specific bacterial taxa.

**Figure 1:**
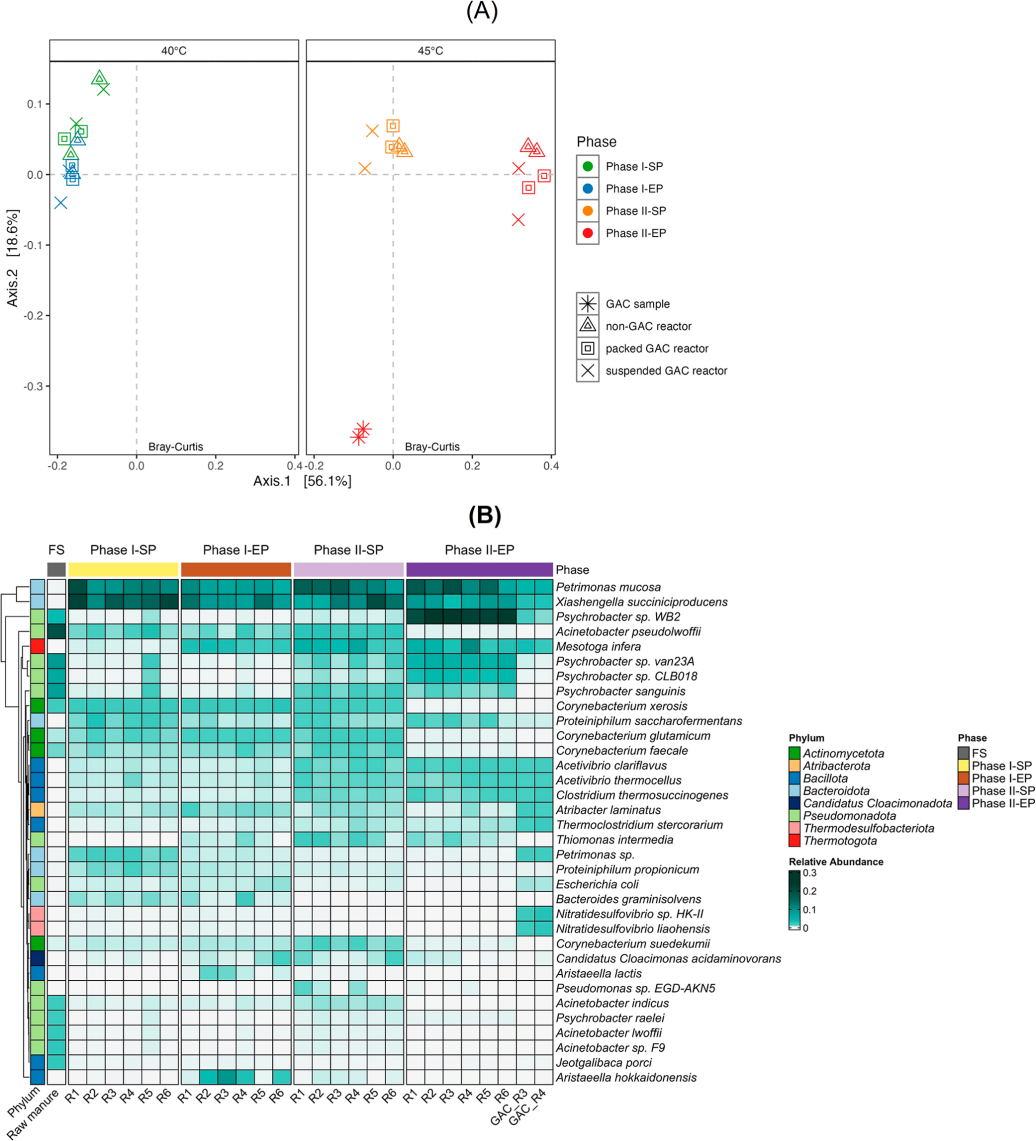
Diversity and composition of the bacterial community. (A) PCoA of the bacterial community at the species level. (B) Top 10 dominant bacterial species in each sample. Phase I-SP: stable, start of phase I; Phase I-EP: stable, end of phase I; Phase II-SP: transition, start of phase II; Phase II-EP: recovery and adaptation, end of phase II; R1 & R2: non-GAC reactors; R3 & R4: packed GAC reactors; R5 & R6: suspended GAC reactors.

In phase I-EP, the six reactors were dominated by phyla *Bacteroidota* (26.99–38.20%), *Bacillota* (18.97–31.15%), *Pseudomonadota* (16.56–22.11%), *Actinomycetota* (12.09–14.49%), *Thermodesulfobacteriota* (2.15–2.82%), and *Thermotogota* (1.45–3.59%) (Figure S2, Supporting Information 1). This was markedly distinct from the raw manure, which was dominated by *Pseudomonadota* (77.93%). After the first 20 days of transition to 45 °C (phase II-SP), a slight shift in the relative abundance of *Pseudomonadota* (19.58–28.09%) and *Thermotogota* (1.70–7.20%) was observed in all reactors; however, the alpha diversity (Shannon index and Pielou’s evenness) remained analogous to that of phase I-EP (Figure S3, Supporting Information 1). Prolonged operation at 45 °C ultimately decreased the alpha diversity, particularly in the non-GAC group. At this point (phase II-EP), *Pseudomonadota* (39.94–47.97%) became the core phylum, and further enrichment of *Thermotogota* (2.15–11.25%) occurred. Members of the phylum *Thermotogota* possess thermostable enzymes and have been found to thrive in hyper-mesophilic and thermophilic digesters.^10,43,44^ In the GAC biofilms, *Bacillota* and *Pseudomonadota* were predominant, jointly accounting for 52–56% of the total bacterial abundance.

At the species level, *Petrimonas mucosa* (5.88–19.72%) and *Xiashengella succiniciproducens* (3.66–21.68%) were notably abundant in the reactors during phases I and II (Figure 1B). Although sparsely represented in the raw manure (<0.1%), *P. mucosa* maintained a consistent relative abundance before and after temperature change. This may be explained by the broad growth temperature of *P. mucosa*, which ranges between 20 and 50 °C with an optimum of 45 °C.^45^ *X. succiniciproducens*, on the other hand, has a growth range of 25–42 °C^46^ and declined in abundance during phase II. As the name suggests, this newly delineated bacterium can produce succinate from the fermentation of monosaccharides.^46^

Thermotolerant and thermophilic species such as *Mesotoga infera*, *Acetivibrio clariflavus*, *Acetivibrio thermocellus*, and *Clostridium thermosuccinogenes* demonstrated a clear preference for growth at 45 °C compared to 40 °C. Likewise, certain strains of *Psychrobacter*, including *P*. sp. *WB2*, *P*. sp. *van23A*, and *P*. sp. *CLB018* were more evident in the digestate after the increase in temperature to 45 °C. These strains were rarely detected during phase I despite having high relative abundance in the raw manure. Among them, *P.* sp. *WB2* was substantially enriched in phase II-EP (19.72–23.87%), becoming the most dominant bacterial species in the six reactors. This trend may not necessarily be driven by active growth, but rather a result of the extensive decrease in microbial diversity, which inadvertently increased the rate of detection of immigrant organisms present in high proportions in the raw manure. Most members of the genus *Psychrobacter* are considered obligate aerobes and psychrophiles, yet some species and strains have been found to tolerate anaerobic conditions and grow at mesophilic temperatures.^47–49^ Li et al.^50^ reported the dominance of *Psychrobacter* species and their potential involvement in the hydrolysis of fat in a mesophilic anaerobic digester fed with pig manure, kitchen waste, and excess sludge.

The dominant bacterial species associated with the GAC surface include *P. mucosa* (4.86–5.18%), *X. succiniciproducens* (2.77–3.04%), *M. infera* (1.84–3.22%), *Nitratidesulfovibrio* sp*. HK-II* (1.86–2.70%), and *Nitratidesulfovibrio liaohensis* (1.58–2.27%). *N*. sp*. HK-II* and *N. liaohensis* are sulfate-reducing bacteria (SRB), and their selective enrichment in the GAC biofilm can be justifiably linked to electroactivity and syntrophic interaction with methanogens. *N.* sp*. HK-II* was originally isolated from the anode of a microbial fuel cell and found to synthesize biogenic mackinawite (conductive iron sulfide mineral) under anoxic conditions in the presence of sulfate and ferric iron.^51^ Akin to the present study, Zhang et al.^14^ noted the enrichment of SRB in GAC biofilm during the AD of wastewater using an up-flow anaerobic sludge blanket reactor. The authors surmised that synergy or competition between SRB and methanogens may depend on the availability of sulfate.

#### 3.2.2 Archaeal Population

The archaeal community evolved temporally due to temperature increase, but compared to the bacterial community, its diversity and composition changed at a slower rate (Figure S4A, Supporting Information 1). This finding was reflected in the tight clustering of the reactors in phases I-SP, I-EP, and II-SP, and subsequent divergence in phase II-EP. The lagged evolution of archaea can be ascribed to their slower growth rate and longer doubling time.^52^ PERMANOVA and PERMDISP support this inference, underscoring that temperature change exerted a greater effect on the bacterial community than on the archaeal community (PERMANOVA: adonis2, R^2^ = 0.36, F = 4.05, p = 0.0013; PERMDISP: p = < 0.01), whereas GAC exerted a greater effect on the archaeal community than on the bacterial community (PERMANOVA: adonis2, R^2^ = 0.10, F = 1.69, p = 0.14; PERMDISP: p = 0.46).

Figure S5 shows the composition of the archaeal community at the phylum level during phases I and II (Supporting Information 1). The phylum *Methanobacteriota* solely accounted for 99.69–99.98% of the total archaeal population in the six reactors. The raw manure, in contrast, harbored *Methanobacteriota* (81.60%) as well as phyla *Thermoproteota* (11.92%), *Nitrososphaerota* (4.12%), and *Thermoplasmatota* (1.94%). At the species level, *Methanosarcina mazei* and *Methanobacterium formicicum* dominated in phase I, with varying representation across reactor designations (Figure S4B, Supporting Information 1). Interestingly, *M. mazei* occurred in higher abundance in the suspended GAC (89.75 ± 1.02%) and non-GAC (89.34 ± 3.30%) reactors than in the packed GAC reactors (78.20 ± 6.52%), while *M. formicicum* was more enriched in the packed GAC reactors (13.88 ± 0.58%) than in the suspended GAC (6.00 ± 1.24%) and non-GAC (5.78 ± 1.86%) reactors. *M. mazei* is a metabolically versatile archaeon capable of utilizing acetate, H_2_/CO_2_, and methylated compounds for methane production.^53,54^ *M. formicicum*, on the other hand, is primarily associated with hydrogenotrophic methanogenesis.^55^ The preferential enrichment of *M. formicicum* in the packed GAC reactors may be configuration dependent. It is plausible that by enhancing biomass retention and enabling the homogeneous distribution of GAC, the packed GAC configuration provided a more structured environment for the establishment of syntrophic partnerships. In phase II-EP, the dominant archaea shifted to *Methanosarcina barkeri* in R1 and R2, and previously underrepresented species, such as *Methanosarcina flavescens*, *Methanosarcina thermophilia*, *Methanosarcina hadiensis*, and *Methanosarcina horonobensis*, markedly increased in abundance in R3, R4, and R6.

The vast proportion of the reads (84–92%) obtained from the GAC biofilm samples were assigned to the archaea domain, suggesting that archaea were tightly bound to the GAC surface and occupied the inner layers, whereas bacteria colonised the periphery. A similar finding was reported in studies that evaluated the spatial organization of bacteria and archaea on GAC surface and in anaerobic granules.^56,57^ In the present study, the GAC biofilm was dominated by *Methanoculleus receptaculi* (52.61–66.19%), a strict hydrogenotrophic methanogen.^58^

### 3.3 Reduction of *E. coli* and Presumptive ARB

The prevalence of indicator pathogens, e.g., *E. coli* and *Salmonella*, is an important metric used to determine the effectiveness of the AD process to ensure the digestate heading to land is safe and fit for purpose. In phase I, the reduction efficiency of viable *E. coli* exceeded 90% in all six reactors (Figure 2). The highest reduction was obtained in the packed GAC reactors (95.70% and 96.24%), followed by the suspended GAC (94.53% and 95.69%) and then the non-GAC (92.77% and 94.24%) reactors. Even without postdigestion pasteurization, the concentration of *E. coli* in all digestate samples complied with the UK’s quality standard for animal byproduct digestate, which stipulates that viable counts should not exceed 3.7 log_10_ CFU/g fresh weight (equivalent to 5–5.1 log_10_ CFU/g dry matter based on the present study) in any one sample or 3 log_10_ CFU/g fresh weight (equivalent to 4.3–4.4 log_10_ CFU/g dry matter based on the present study) in more than two samples.^59^ At 45 °C (phase II), viable *E. coli* fell below detectable levels, highlighting the role of temperature in pathogen inactivation during AD. From the outcome of partial least-squares (PLS) regression, a recent meta-analysis affirmed that temperature exerts more influence on pathogen reduction than pH, hydraulic retention time, and phenotypic traits.^60^ Underlying mechanisms include protein denaturation, enzyme inhibition, cell membrane disruption, and nucleic acid damage.^61,62^

**Figure 2:**
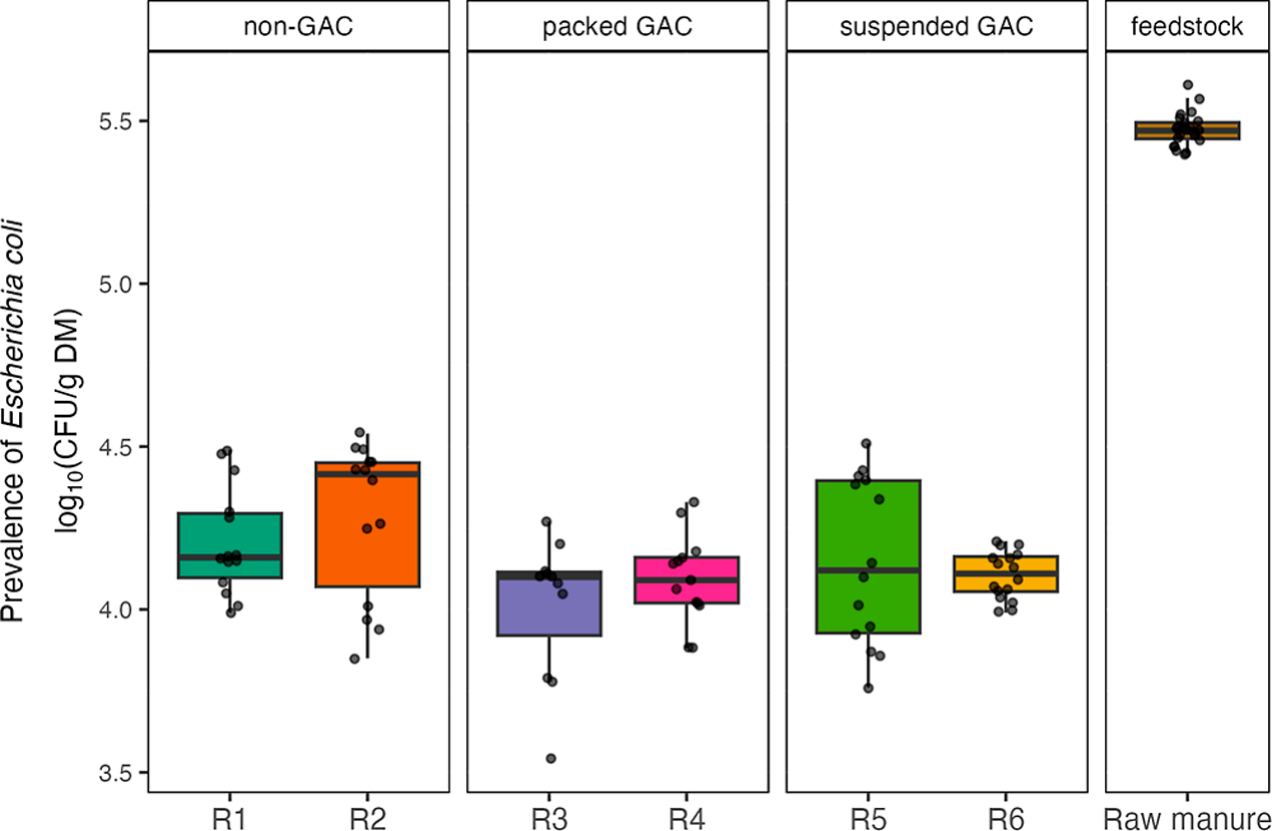
Persistence of viable *E. coli* during phase I. The edges of the box represent the 25th and 75th percentiles, the solid line in the interior depicts the median, and the protruding whiskers correspond to the minimum and maximum values. Counts were below the detection and quantification limit in all six reactors during phase II. CFU: colony-forming units. DM: dry matter.

The reduction of culturable tetracycline and ampicillin-resistant bacteria was measured over multiple time points during phases I and II (Table 2). In both phases, the concentration of tetracycline-resistant bacteria was consistently below the quantification limit (<30 CFU) in all six reactors. This significantly contrasts with the 6.19 log_10_ CFU/g-DM detected in the raw manure; therefore, it can be inferred that AD at 40 and 45 °C effectively suppressed the growth of bacteria resistant to 25 μg/mL tetracycline. In the case of ampicillin-resistant bacteria, count values obtained for the suspended GAC reactors (R5 and R6) during phases I and II were below the quantification limit. For the other two experimental groups, count values were conflicting during phase I, with R2 (non-GAC) and R4 (packed GAC) having marginally higher levels of ampicillin-resistant bacteria than the raw manure. An increase in the log reduction of ampicillin-resistant bacteria was achieved in the reactors during phase II, indicating that higher temperature, in this instance, was more beneficial for the reduction of ampicillin-resistant bacteria. This trend is likely a reflection of the sensitivity of some ampicillin-resistant bacteria to thermal stress, which can engender shifts in community structure by selectively inhibiting less tolerant populations.^63^ In addition to cell death, thermal stress can trigger a state whereby cells are viable but nonculturable (VBNC).^64^

**Table 2:** Counts of Presumptive Tetracycline and Ampicillin-Resistant Bacteria During Phases I and II

phase	designation	reactor ID	tetracycline (log_10_ CFU/g-DM)^a^ ^,^ ^b^	ampicillin (log_10_ CFU/g-DM)^a^ ^,^ ^b^
I (40 °C)	non-GAC	R1	BQL	5.68 ± 0.15
		R2	BQL	5.95 ± 0.073
	packed GAC	R3	BQL	5.33 ± 0.025
		R4	BQL	5.99 ± 0.078
	suspended GAC	R5	BQL	BQL
		R6	BQL	BQL
II (45 °C)	non-GAC	R1	BQL	5.66 ± 0.083
		R2	BQL	5.25 ± 0.18
	packed GAC	R3	BQL	5.27 ± 0.099
		R4	BQL	5.18 ± 0.05
	suspended GAC	R5	BQL	BQL
		R6	BQL	BQL
–	raw manure	–	6.19 ± 0.076	5.72 ± 0.075

a Values are expressed as mean ± standard deviation (*n* ≥ 2).

b BQL: below quantification limit; CFU: colony-forming units; DM: dry matter.

### 3.4 Distribution and Fate of ARGs

#### 3.4.1 Total Abundance of ARGs

The characterization of the resistome profiles of the raw manure, digestate, and GAC biofilm samples revealed the predominance of genes encoding resistance to macrolide-lincosamide-streptogramin (MLS), aminoglycoside, bacitracin, polymyxin, multidrug, tetracycline, beta-lactam, streptothricin, sulfonamide, and vancomycin (Figure 3). Several of these antibiotic classes, notably beta-lactam, tetracycline, and aminoglycoside, are actively sold and administered to dairy cattle in the UK, and their resistance genes are frequently detected in cattle manure.^25,65,66^ Although polymyxin antibiotics are used sparingly in cattle, the genes associated with polymyxin resistance were prevalent in this study. From a public health standpoint, this finding is concerning because polymyxins, e.g., colistin, are considered “highest priority critically important antibiotics” (HP-CIAs, also termed “last resort antibiotics”).^25^ Acquired resistance to colistin severely impedes the treatment of life-threatening multidrug-resistant infections caused by Gram-negative bacteria such as carbapenemase-producing *Enterobacteriaceae*.^67,68^

**Figure 3:**
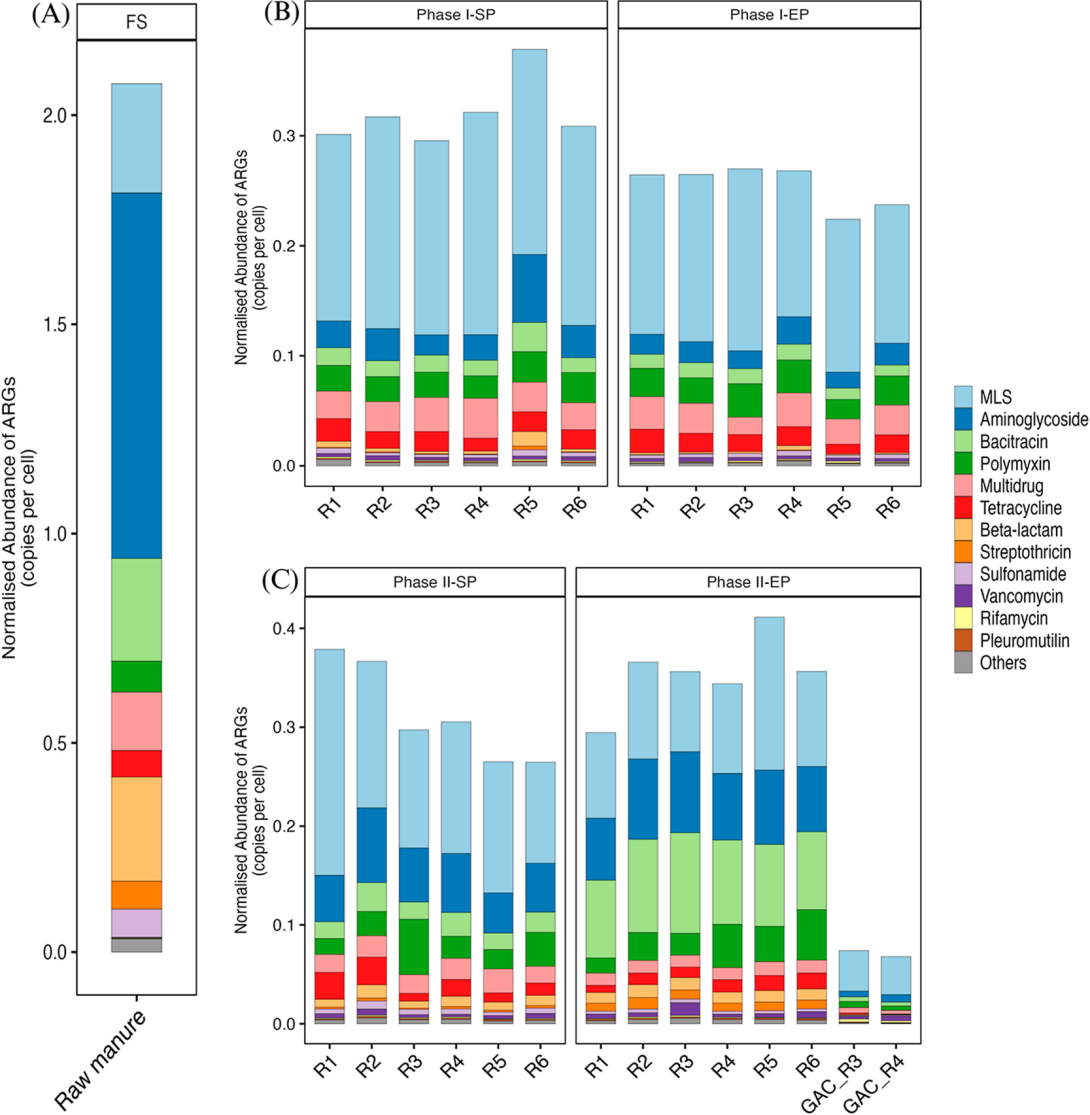
Normalized abundance of ARGs according to resistance class. (A) Raw manure. (B) Phase I. (C) Phase II. R1 & R2: non-GAC reactors; R3 & R4: packed GAC reactors; R5 & R6: suspended GAC reactors.

A marked difference in ARG levels was obtained before and after AD. Relative to the raw manure, the total abundance of ARGs in the reactors during phases I and II decreased by 80.18–89.20%, demonstrating that AD at hyper-mesophilic temperatures (40 and 45 °C) can reduce ARGs in cattle manure to a high degree. This aligns with the 91–92% reduction efficiency reported in the study of a full-scale biogas plant operating at 44 °C and feeding a blend of maize and cattle manure.^10^ It should be noted, however, that while shotgun metagenomics was deployed in both studies, comparisons may be skewed by differences in quantification unit (copies per cell vs RPKM), analysis approach (read-based vs contig-based), and sequencing depth. In phase I-SP, the reduction efficiency of total ARGs ranged from 84.72 to 85.48% in the non-GAC reactors, 84.51 to 85.76% in the packed GAC reactors, and 81.76 to 85.13% in the suspended GAC reactors. These values increased slightly across all six reactors in phase I-EP, with the suspended GAC reactors (88.56–89.20%) attaining higher efficiency than the non-GAC (87.24–87.26%) and packed GAC (86.99–87.08%) reactors. The change of temperature to 45 °C (phase II-SP) decreased the reduction efficiency of aminoglycoside, beta-lactam, and bacitracin resistance classes, and increased that of tetracycline and multidrug. During this phase, the suspended GAC reactors (87.23–87.25%) maintained nearly 2 percentage points higher reduction efficiency than the packed GAC reactors (85.28–85.67%) and about 5 percentage points higher reduction efficiency than the non-GAC reactors (81.75–82.33%). Extended operation at 45 °C (phase II-EP) led to further decline in the reduction efficiency of bacitracin and streptothricin resistance classes. Compared to the digestate samples, ARG levels in the GAC biofilms were significantly lower. This is likely connected to the markedly low diversity of the microbial community associated with the GAC biofilms, as highlighted by the alpha diversity indices (Figure S3, Supporting Information 1). Moreover, the GAC biofilms were dominated by methanogenic archaea, which rarely harbor ARGs.

Overall, the results show that the total abundance of ARGs in the reactors was lower in phase I (40 °C) compared to phase II (45 °C). Temperature transition substantially promoted the reduction of some resistance classes (MLS, tetracycline, and multidrug) but diminished the reduction of others (bacitracin, aminoglycoside, beta-lactam, and streptothricin). This outcome could be intrinsically tied to the growth, tolerance, and adaptation of specific ARG host taxa. For example, ARGs harbored by bacterial taxa that thrive at 45 °C are bound to be differentially abundant and may potentially spread through horizontal gene transfer (HGT) as the hosts proliferate. In the same vein, the growth of temperature-sensitive hosts could be suppressed, leading to the reduction of some ARGs.

#### 3.4.2 Prevalent ARG Subtypes

The ARGs detected in at least 50% of the reactor samples were selected for differential abundance analysis to assess shifts in abundance under different operating conditions (Supporting Information 2). The result revealed that several ARGs, totalling 94, differed significantly between phases (DESeq2, Wald test, 40 °C vs 45 °C, adjusted *p* < 0.05); however, no significant difference was found between reactor configurations in either phase I or II (DESeq2, Likelihood Ratio Test, non-GAC vs packed GAC vs suspended GAC, adjusted *p* > 0.05).

In the raw manure, the most abundant ARGs comprised *APH­(3′)-Ib*, *APH­(6)-Id*, and *aadS* (aminoglycoside); *bacA* (bacitracin); *lnu­(H)* (MLS); *ugd* (polymyxin); *SAT-2* (streptothricin); and *sul2* (sulfonamide) (Figure 4). Among them, *APH­(3′)-Ib*, *APH­(6)-Id*, *bacA*, *SAT-2*, and *sul2* followed a similar trajectory in all reactors, exhibiting significantly higher reduction in phase I compared to phase II (adjusted *p* < 0.05). Conversely, *lnu­(H)* and *aadS* resistance genes exhibited greater reduction in phase II, suggesting that the removal of the host population of these genes may have been favored by temperature shift. The *ugd* gene responded differently, reducing in abundance but showing no notable variation between phases I and II. This gene encodes the UDP-d-glucose dehydrogenase enzyme required for the modification of the outer membrane lipopolysaccharide structure, which ultimately decreases the binding affinity for polymyxins.^69,70^

**Figure 4:**
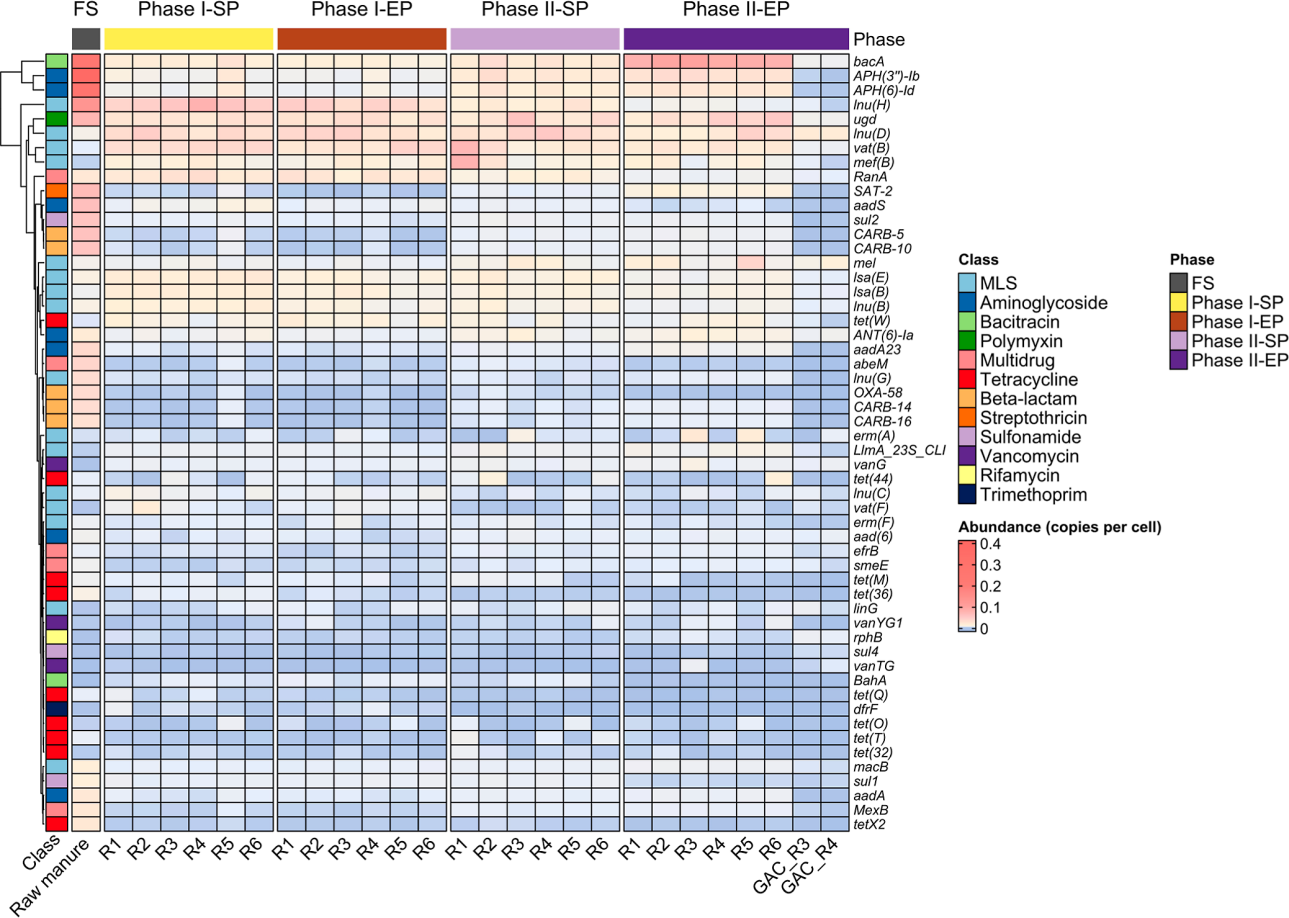
Top 20 most abundant ARGs in the raw manure, digestate, and GAC biofilm samples. R1 & R2: non-GAC reactors; R3 & R4: packed GAC reactors; R5 & R6: suspended GAC reactors.

Beta-lactam resistance genes, including *CARB-5*, *CARB-10*, *CARB-14*, and *CARB-16*, also occurred in high proportions in the raw manure and were reduced in phase I more than in phase II. In addition, the trimethoprim resistance gene, *dfrF*, was present in the raw manure and all six reactors in phase I but was undetected in phase II. This gene was reported to be responsible for high-level trimethoprim resistance in clinical pathogens like *Enterococcus faecalis* and *Streptococcus pyogenes*.^71,72^ It is horizontally transmissible and has been found on integrative conjugative elements (ICEs) in strains of Group A *Streptococcus*.^71,73^

Some ARGs detected at low levels in the raw manure were enriched in the reactors in phases I and II. These include the MLS resistance genes *lnu­(B)*, *lnu­(D)*, *lsa­(B)*, *lsa­(E)*, *mef­(B)*, *mel*, and *vat­(B)*, as well as the tetracycline resistance gene *tet­(W)*. The *lnu­(B)* and *tet­(W)* genes are among the 37 ARGs listed as high risk by the World Health Organisation on account of clinical significance and widespread occurrence in MGEs.^74^ Their enrichment in the digestate raises concerns due to the serious threat they pose to human health. Other high-risk ARGs like *sul1* (sulfonamide) and *tet­(M)* (tetracycline) were highly reduced during phase II.

#### 3.4.3 Correlation Between ARGs and Bacterial Community Structure

To contextualise the dynamics of ARGs, the relationship between ARGs and the bacterial community was examined. The PCoA ordination of the resistome resonated with that of the bacterial community (Figure S6, Supporting Information 1). All six reactors diverged temporally between phases, indicating that temperature was a dominant factor shaping the composition of the resistome (PERMANOVA: adonis2, R^2^ = 0.70, F = 15.12, p = 0.0001; PERMDISP: p = < 0.05). By contrast, a significant difference was not observed between reactor configurations (non-GAC, packed GAC, and suspended GAC), indicating that GAC played a limited role (PERMANOVA: adonis2, R^2^ = 0.02, F = 0.54, p = 0.78; PERMDISP: p = > 0.05). To further explore whether the variation of ARGs can be explained by shifts in the bacterial community structure, the Mantel test and Procrustes analysis were performed. The outcome of both analyses revealed a significant correlation between the resistome and the bacterial community (Mantel test: r = 0.65, p = 0.001; Procrustes: M^2^ = 0.74, correlation = 0.51, p = 0.001, Figure 5). Collectively, the PERMANOVA, Mantel test, and Procrustes analysis affirmed that the response of the ARG host population to temperature change was a major determinant of the fate of ARGs.

**Figure 5:**
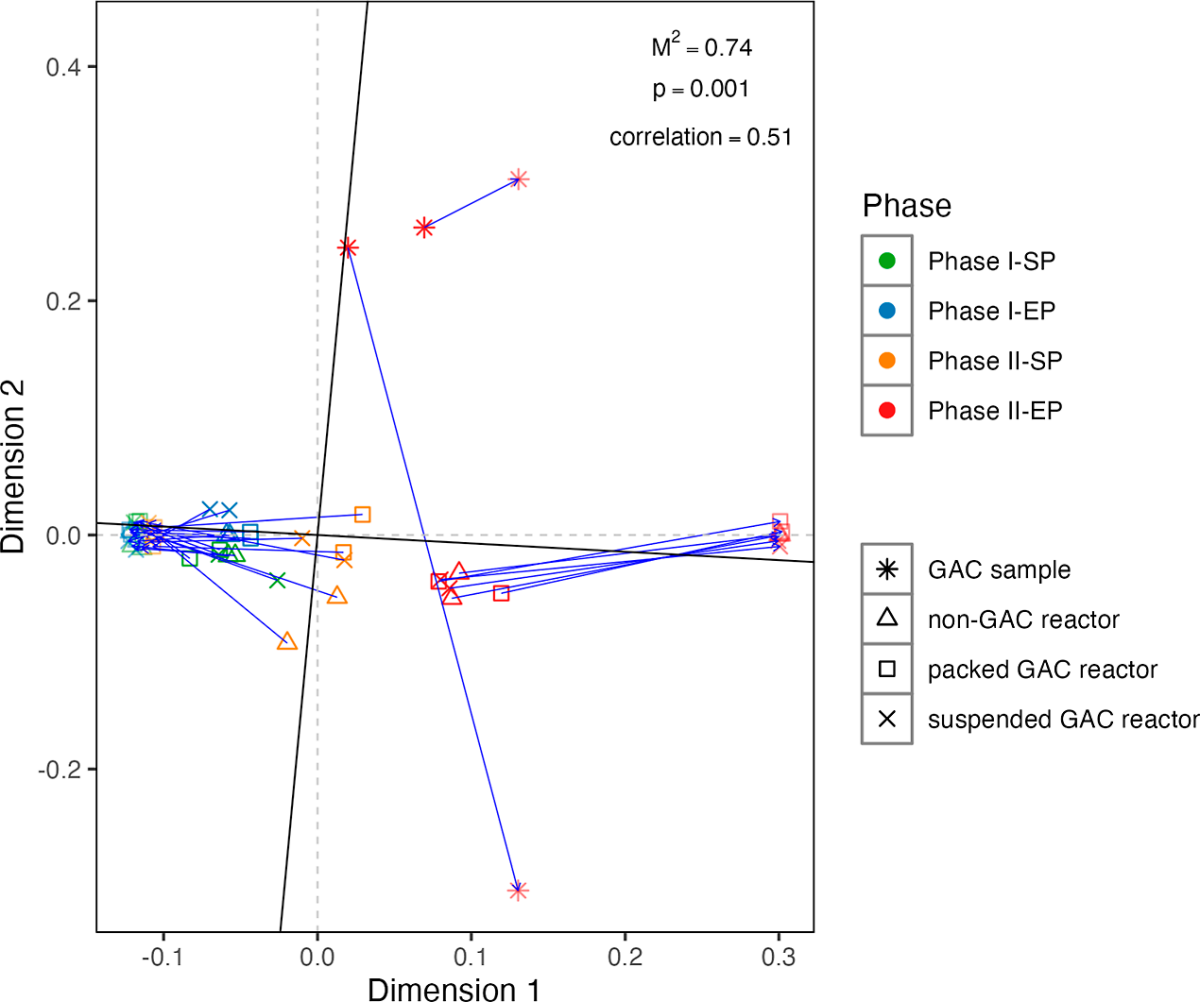
Association of the resistome profile with the bacterial community at the species level.

#### 3.4.4 Potential Hosts of ARGs

Taxonomic annotation of ARG-bearing contigs was undertaken to identify bacterial taxa potentially harboring the prevalent ARG subtypes in the raw manure and reactor samples (Table S2, Supporting Information 1). The MLS resistance gene, *lnu­(D)*, had the broadest host range, occurring in 25 distinct contigs. These contigs were assigned to multiple taxa, including pathogenic (*Streptococcus parasuis*, *Clostridium tetani*, and *Enteroccocus cecorum*), sulfate-reducing (*Desulfitobacterium dichloroeliminans* and *Desulforamulus ferrireducens*), and core fermentative and syntrophic bacteria (*Syntophomonas wolfei*, *Clostridium bornimense*, *A. thermocellus* and *Acetivibro clariflavus*). *A. thermocellus* and *A. clariflavus* were predominant in the reactors; hence, their growth and activity may explain why the *lnu­(D)* gene was substantially more enriched in the reactors compared to the raw manure. *S. wolfei* and *A. thermocellus* were also the potential hosts of *mef­(B)*, another MLS resistance gene that was enriched in the reactors but had low abundance in the raw manure. Of the three most abundant ARGs in the manure and reactors, *APH­(3′)-Ib* and *APH­(6)-Id* (aminoglycoside) were associated with *E. coli*, while *bacA* (bacitracin) was associated with *Nitratidesulfovibrio liahensis*, *Nitratidesulfovibrio vulgaris*, and *Pseudomonas*. Other notable taxa linked to ARGs include *Staphylococcus aureus* (*ANT­(6)-Ia* and *erm­(A)*), *Streptococcus agalactiae* (*vanG* and *vanTG*), *Streptococcus suis* (*vanG* and *lsa­(E)*), *Enterococcus faecium* (*aad­(6)*), *Psychrobacter* (*CARB-5*), *Clostridium perfringens* (*tet­(44)*), and *Clostridium ultunense Esp* (*tet­(T)*). These findings highlight that ARGs are widely distributed across diverse taxa, with functionally relevant guilds serving as key contributors to the persistence of ARGs in cattle manure AD.

#### 3.4.5 Mobility Potential of ARGs

The association of ARGs with MGEs (e.g., insertion sequences, integrons, transposons, integrative conjugative elements, and plasmids) is typically used as a proxy for estimating the potential for the horizontal transfer of resistance determinants in a microbiome. On this basis, the annotation of MGEs was undertaken, revealing the pronounced abundance of transposase (*tnpA*) and insertion sequences (*IS91* and *IS26*) in the raw manure (Figure S7A, Supporting Information 1). Plasmid replication genes (*rep*), conjugative transposons (*Tn916*), and integrase (*intI1* and *intI2*) were also detected, but at comparatively lower levels. The *Tn916* transposon and the class 1 integrase gene *intI1* are important sentinels of HGT and have been implicated in the mobilization and spread of ARGs among diverse commensal and pathogenic bacteria.^75,76^

As shown in Figure S7B,C (Supporting Information 1), the distribution of MGEs in the reactors was analogous to that of ARGs, with higher total abundance observed in phase II (45 °C) than in phase I (40 °C). Relative to the raw manure, the total abundance of MGEs during phase I-EP decreased by 97.92–98.39% in the suspended GAC reactors, 96.06–97.43% in the packed GAC reactors, and 96.30–97% in the non-GAC reactors. This demonstrates that hyper-mesophilic AD can effectively reduce MGEs in cattle manure, thereby mitigating risks of AMR propagation via HGT. A change in the operating temperature, i.e., phase II-EP, enhanced the reduction of *IS91* and integrase but also resulted in the enrichment of *rep* genes and *tnpA* transposase gene to levels marginally above what was recorded in phase I. During phase II-EP, the reduction of total MGEs was comparable in the six reactors, ranging between 88.92 and 91.38%.

To further delineate the ARG-MGE association, contigs were examined for the colocalization of ARGs and MGEs. Of the 128 ARG-bearing contigs screened, three were found to also harbor an MGE (Figure S8, Supporting Information 1). All three contigs were detected in the raw manure and reactor samples. In one of the contigs, *sul2* co-occurred with *tnpA*. This contig was classified as *Acinetobacter pseudolwoffii*. In the other two unclassified contigs, *tet­(A)* co-occurred with *tnpA*, while *sul1* co-occurred with the *qacE*Δ*1* gene, which is commonly associated with class 1 integrons.

## 4 Conclusion

This study explored the impact of different GAC application strategies (suspended and packed) on pathogen and ARG reduction in the AD of cattle manure at 40 and 45 °C. The results revealed that GAC strongly buffered the shock induced by temperature transition but yielded only marginal gains in the reduction of viable *E. coli* (indicator pathogen), ARGs, and MGEs. The packed GAC reactors exhibited slightly better reduction efficiency for *E. coli*, whereas the suspended GAC reactors achieved greater reduction of culturable ampicillin-resistant bacteria, ARGs, and MGEs. It would be worthwhile for future studies to build on these findings by exploring whether the combined application of packed and suspended GAC configurations can be leveraged to further enhance outcomes. Compared to GAC, temperature played a more significant role in shaping the structure of the microbial community, resistome, and mobilome. Higher operating temperature (45 °C) favored the reduction of viable *E. coli*; however, its effect on ARGs and MGEs varied. While the reduction of certain ARGs and MGEs was enhanced at a higher temperature, the reduction of others was diminished. Overall, this study showed that hyper-mesophilic AD (40 and 45 °C) can effectively suppress pathogens and reduce the burden of AMR determinants in cattle manure, regardless of additive application. We highly recommend the integration of culture-based and culture-independent methods for a robust surveillance and risk assessment of AMR in natural and engineered environments.

## Supplementary Material





## Data Availability

The metagenomic sequencing data generated in this study can be accessed via the NCBI Sequence Read Archive under BioProject accession number PRJNA1344414.

## Notes

Supporting Information 1: the characteristics of the raw manure (Table S1), the potential hosts of ARGs (Table S2), the biogas productivity (Figure S1), the bacterial community composition (Figure S2), the alpha diversity (Figure S3), the archaeal community composition (Figures S4 and S5), the PCoA of the resistome (Figure S6), the abundance of MGEs (Figure S7), and the co-occurrence of ARGs and MGEs in contigs (Figure S8) (PDF).
Supporting Information 2: outputs of differential abundance analysis performed using DESeq2 (XLSX).
