# Supplementary material for: Dynamics of the Microbiome and Antibiotic Resistome in Hyper-Mesophilic Anaerobic Digestion of Cattle Manure Assisted with Granular Activated Carbon
Source: ACS Environ Au. 2026 Feb 18;6(3):435–48. doi: 10.1021/acsenvironau.5c00239 (PMC13195469; doi:10.1021/acsenvironau.5c00239)
Supplement: Supplementary file 1 [file vg5c00239_si_001.pdf]

Supporting Information

Dynamics of the microbiome and antibiotic resistome in hyper-mesophilic anaerobic digestion of cattle manure assisted with granular activated carbon.

Mac-Anthony Nnorom [a], Bang Du [a], Zhufang Wang [a], Zilin Tian [a], Rupert Hough [b], Lisa Avery [b], Devendra Saroj [a], Bing Guo [a, *]

[a] School of Engineering, University of Surrey, Guildford GU2 7XH, United Kingdom.

[b] The James Hutton Institute, Craigiebuckler, Aberdeen AB15 8QH, United Kingdom.

[*] Corresponding author: Bing Guo (b.guo@surrey.ac.uk)

TABLES

Table S1. Characteristics of the raw cattle manure.

**Parameters**[1]	**Unit**	**Value**
pH 1:6 [Fresh]		7.72
Oven Dry Matter	%	23.57
Total Nitrogen	% w/w	1.34
Ammonium Nitrogen	mg/kg	316
Nitrate Nitrogen	mg/kg	< 10
Total Phosphorus (P)	% w/w	0.503
Total Potassium (K)	% w/w	0.251
Total Magnesium (Mg)	% w/w	0.591
Total Sulphur (S)	% w/w	0.217
Total Copper (Cu)	mg/kg	123
Total Zinc (Zn)	mg/kg	278
Total Sodium (Na)	% w/w	0.199
Total Calcium (Ca)	mg/kg	19197
Total Organic Carbon	%	44.9
Conductivity 1:6 [Fresh]	µS/cm	1008
Total Lead (Pb)	mg/kg	< 1
Total Cadmium (Cd)	mg/kg	0.274
Total Mercury (Hg)	mg/kg	< 0.1
Total Nickel (Ni)	mg/kg	3.18
Total Chromium (Cr)	mg/kg	3.88

[1] Dry matter basis

Table S2. Potential hosts of ARGs

**Gene** [1]	**Taxonomy**	**Resistance class**	**Weight** [2]
aad(6)	*Enterococcus faecium*	Aminoglycoside	1
aad(6)	*Sinanaerobacter sp.*	Aminoglycoside	1
aadS	*Chryseobacterium*	Aminoglycoside	1
aadS	*Chryseobacterium suipulveris*	Aminoglycoside	1
aadS	*Myroides odoratimimus*	Aminoglycoside	1
ANT(6)-Ia	*Desemzia incerta*	Aminoglycoside	2
ANT(6)-Ia	*Desulfofarcimen acetoxidans*	Aminoglycoside	1
ANT(6)-Ia	*Staphylococcus aureus*	Aminoglycoside	1
ANT(6)-Ia	*Acetivibrio clariflavus*	Aminoglycoside	1
APH(3'')-Ib	*Escherichia coli*	Aminoglycoside	1
APH(6)-Id	*Escherichia coli*	Aminoglycoside	1
bacA	*Nitratidesulfovibrio liaohensis*	Bacitracin	1
bacA	*unclassified Pseudomonas*	Bacitracin	1
bacA	*Nitratidesulfovibrio vulgaris*	Bacitracin	1
CARB-5	*Psychrobacter sanguinis*	Beta-lactam	1
CARB-5	*Psychrobacter maritimus*	Beta-lactam	1
erm(A)	*Streptococcus pyogenes*	MLS	1

erm(A)	*Acetivibrio saccincola*	MLS	1
erm(A)	*Staphylococcus aureus*	MLS	1
erm(A)	*Staphylococcaceae*	MLS	1
erm(F)	*Xiashengella succiniciproducens*	MLS	1
linG	*Escherichia coli*	MLS	1
linG	*Trichlorobacter lovleyi SZ*	MLS	1
linG	*Nisaea sp.*	MLS	1
lnu(C)	*Faecalibacterium prausnitzii*	MLS	1
lnu(C)	*Roseburia sp.*	MLS	1
lnu(C)	*Clostridia*	MLS	1
lnu(D)	*Desulfitobacterium dichloroeliminans*	MLS	1
lnu(D)	*Desulforamulus ferrireducens*	MLS	4
lnu(D)	*Clostridium bornimense*	MLS	1
lnu(D)	*Acetivibrio thermocellus*	MLS	1
lnu(D)	*Bacillota*	MLS	1
lnu(D)	*Caldifermentibacillus hisashii*	MLS	2
lnu(D)	*Clostridia*	MLS	1
lnu(D)	*Jeotgalibaca porci*	MLS	1
lnu(D)	*Bacilli*	MLS	1
lnu(D)	*Phosphitispora sp.*	MLS	2
lnu(D)	*Kurthia sp.*	MLS	1
lnu(D)	*Clostridium tetani*	MLS	1
lnu(D)	*Streptococcus parasuis*	MLS	1
lnu(D)	*Enterococcus cecorum*	MLS	1
lnu(D)	*Proteiniclasticum sp.*	MLS	1
lnu(D)	*Acetivibrio clariflavus*	MLS	1
lnu(D)	*Clostridium sp.*	MLS	1
lnu(D)	*Syntrophomonas wolfei subsp. wolfei str. Goettingen G311*	MLS	3
lnuG	*Bacilli*	MLS	2
lsa(B)	*Lysinibacillus sp.*	MLS	1
lsa(E)	*Enterococcus cecorum*	MLS	1
lsa(E)	*Terrisporobacter petrolearius*	MLS	1
lsa(E)	*Streptococcus suis*	MLS	1
mef(B)	*Escherichia coli*	MLS	2
mef(B)	*Thermoclostridium stercorarium*	MLS	1
mef(B)	*Syntrophomonas wolfei subsp. wolfei str. Goettingen G311*	MLS	1
mef(B)	*Acetivibrio thermocellus*	MLS	1
mef(B)	*Gammaproteobacteria*	MLS	1
mel	*Clostridium kluyveri*	MLS	1
mel	*Terrisporobacter petrolearius*	MLS	1
mel	*Clostridium sp.*	MLS	1
mel	*Anaerocolumna sedimenticola*	MLS	1
smeE	*Stenotrophomonas maltophilia*	Multidrug	2
sul1	*Bacteria*	Sulfonamide	1
sul2	*Acinetobacter pseudolwoffii*	Sulfonamide	1
tet(44)	*Clostridium perfringens*	Tetracycline	1
tet(36)	*Chryseobacterium sp.*	Tetracycline	1

tet(36)	*Flavobacteriales*	Tetracycline	1
tet(O)	*Roseburia intestinalis*	Tetracycline	1
tet(Q)	*Parabacteroides distasonis*	Tetracycline	1
tet(T)	*Clostridium tetani*	Tetracycline	1
tet(T)	*Clostridium ultunense Esp*	Tetracycline	1
tet(W)	*Desulfovibrionaceae*	Tetracycline	1
tet(W)	*Lachnospiraceae*	Tetracycline	1
vanG	*Thermocaproicibacter melissae*	Vancomycin	1
vanG	*Clostridia*	Vancomycin	1
vanG	*Streptococcus agalactiae*	Vancomycin	2
vanG	*Streptococcus suis*	Vancomycin	1
vanTG	*Thermocaproicibacter melissae*	Vancomycin	1
vanTG	*Streptococcus agalactiae*	Vancomycin	1

[1] limited to the top 20 most abundant ARGs in the raw manure, digestate, and GAC biofilm samples.

[2] corresponds to the number of individual contigs annotated as the same ARG-host pair.

FIGURES

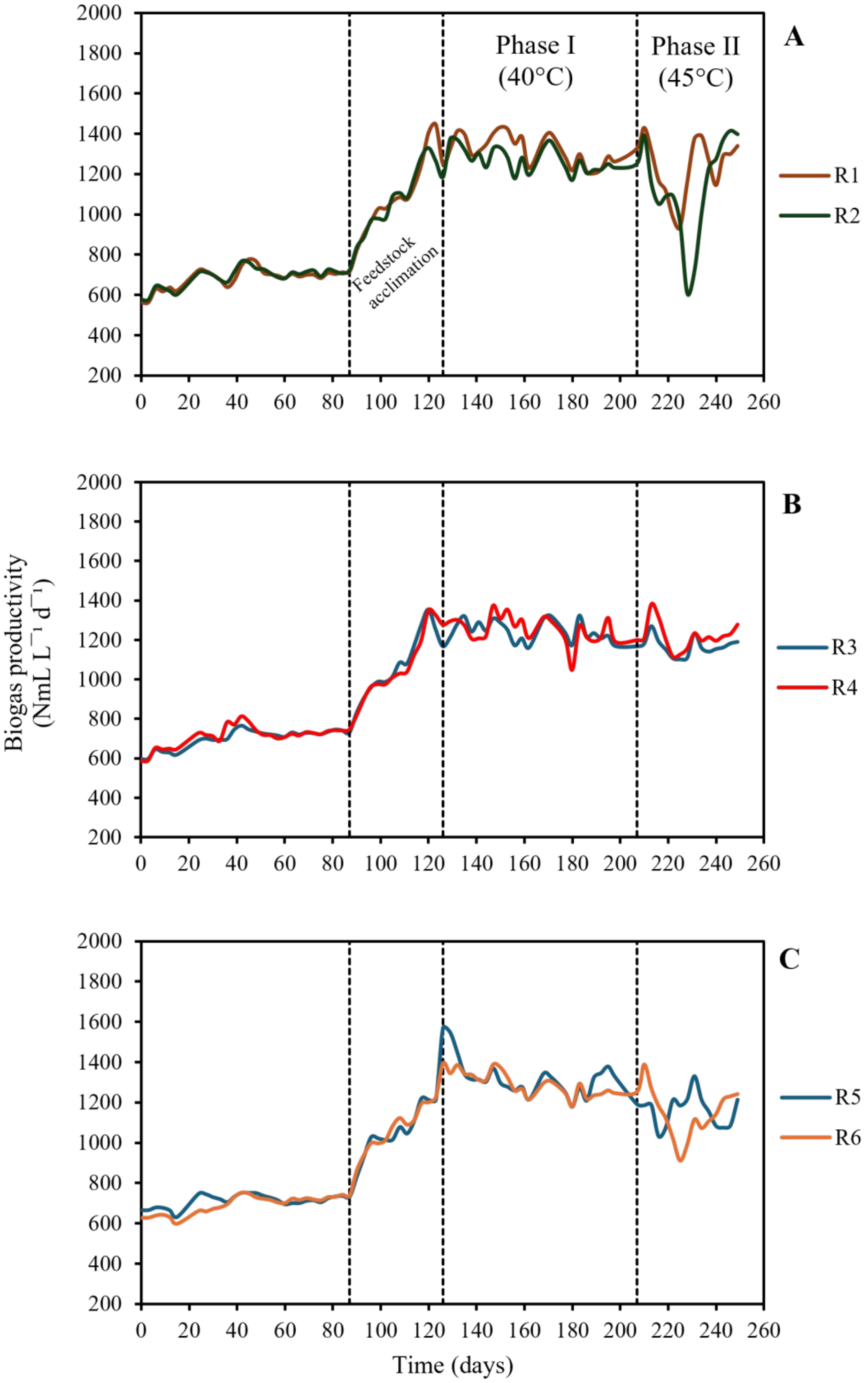

Figure S1. Long-term biogas productivity. (A) non-GAC. (B) packed GAC. (C) suspended GAC.

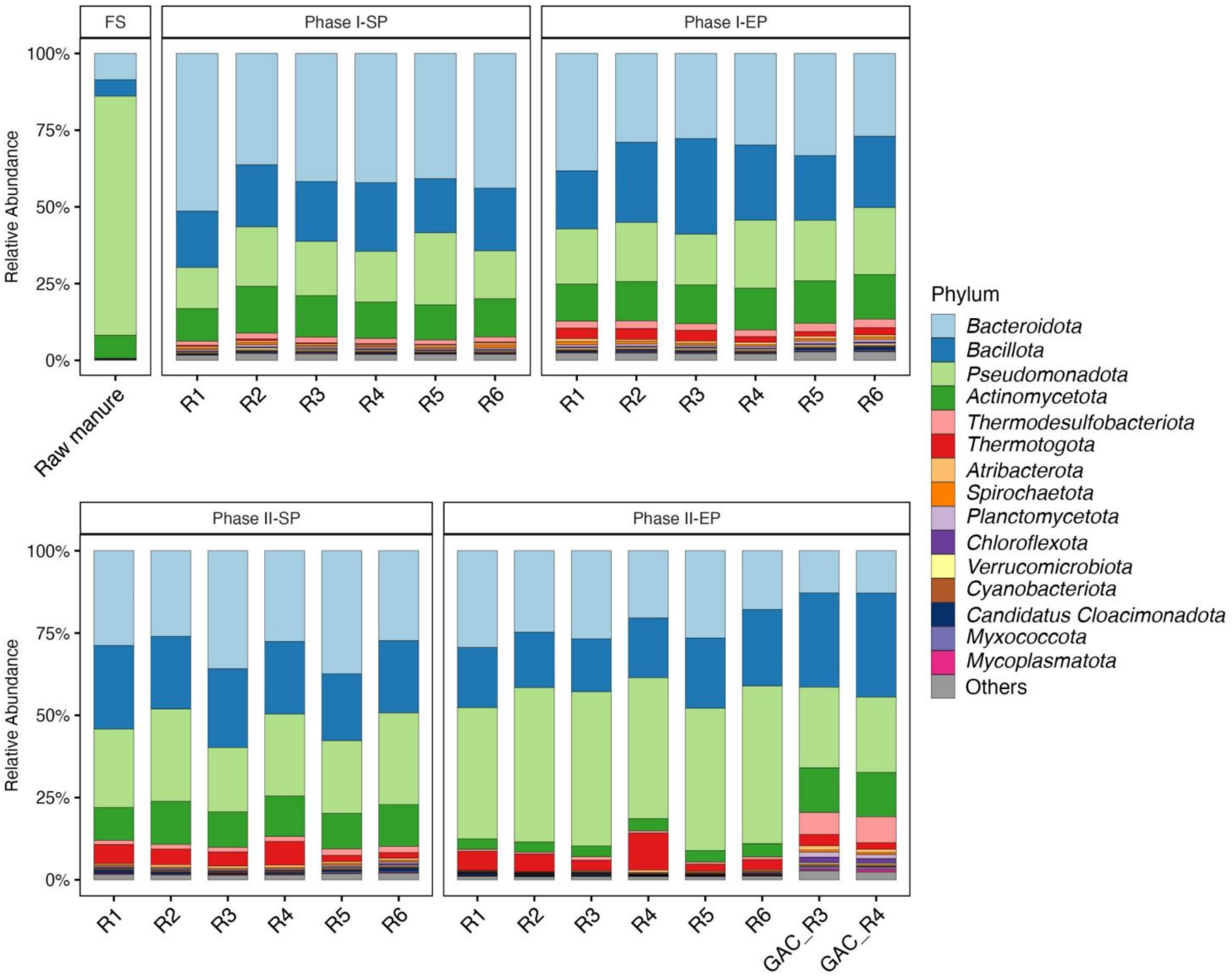

Figure S2. Bacterial community composition at the phylum level.

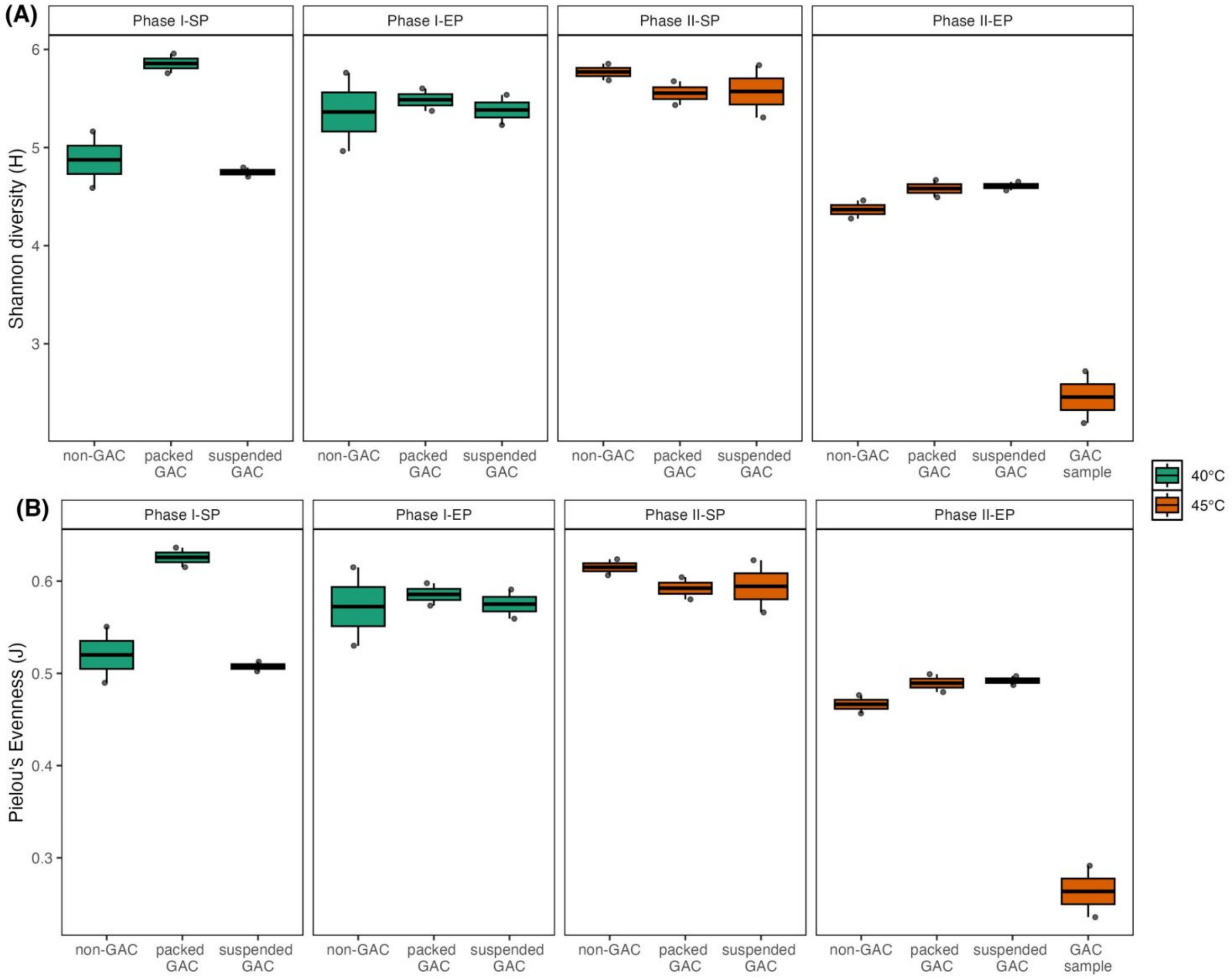

Figure S3. The alpha diversity of the microbial community during the different experimental phases. (A) Shannon diversity. (B) Pielou's evenness.

(A)

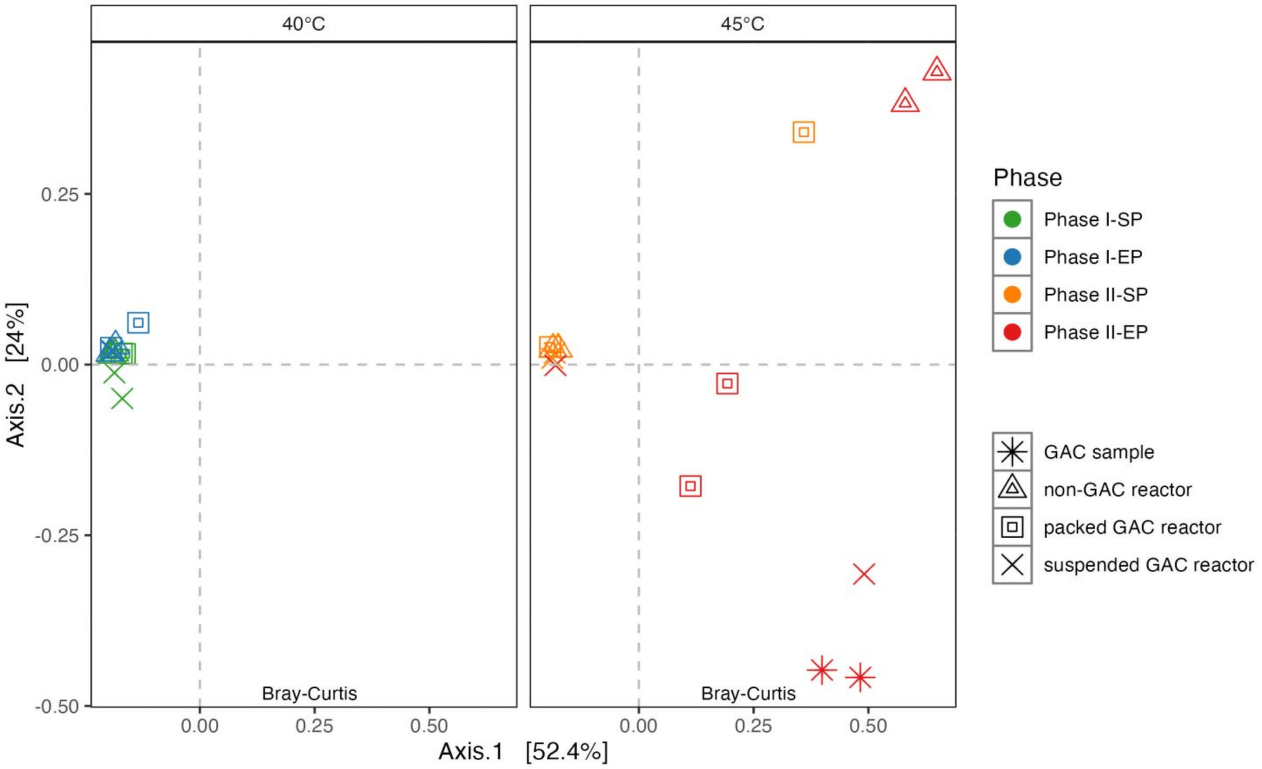

(B)

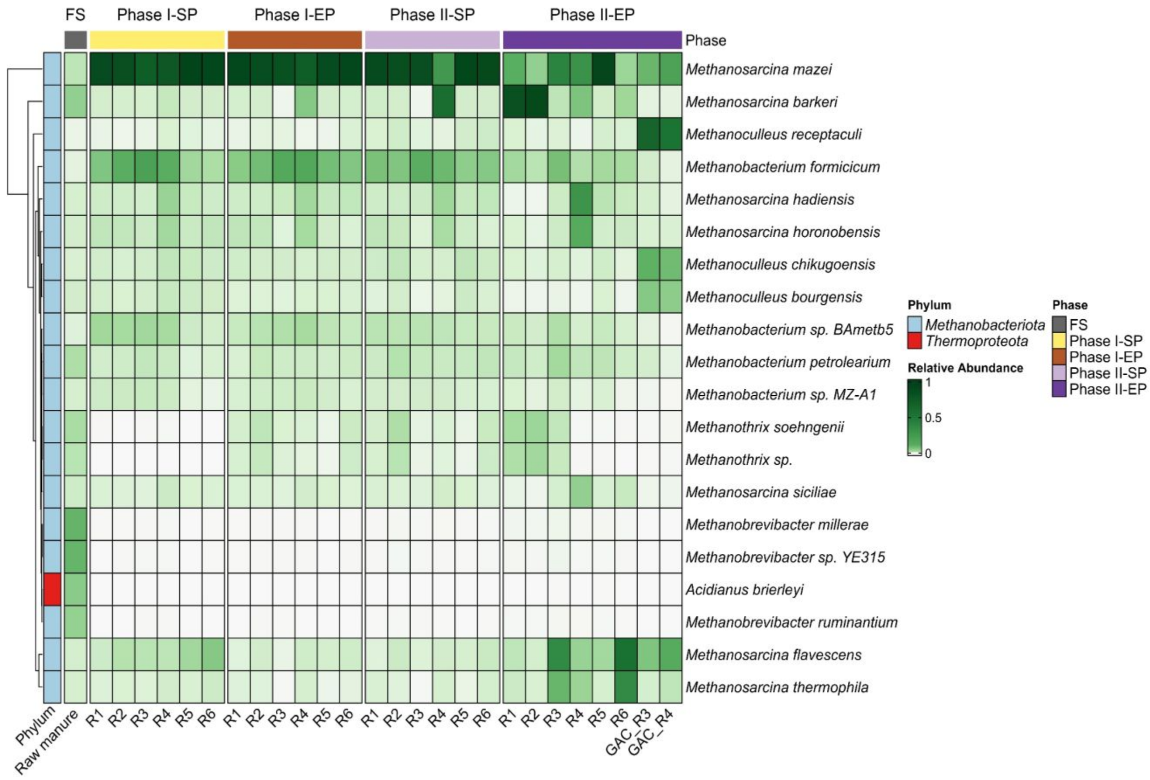

Figure S4. The diversity and composition of the archaeal community. (A) PCoA of the archaeal community at the species level. (B) Top 5 dominant archaeal species in each sample.

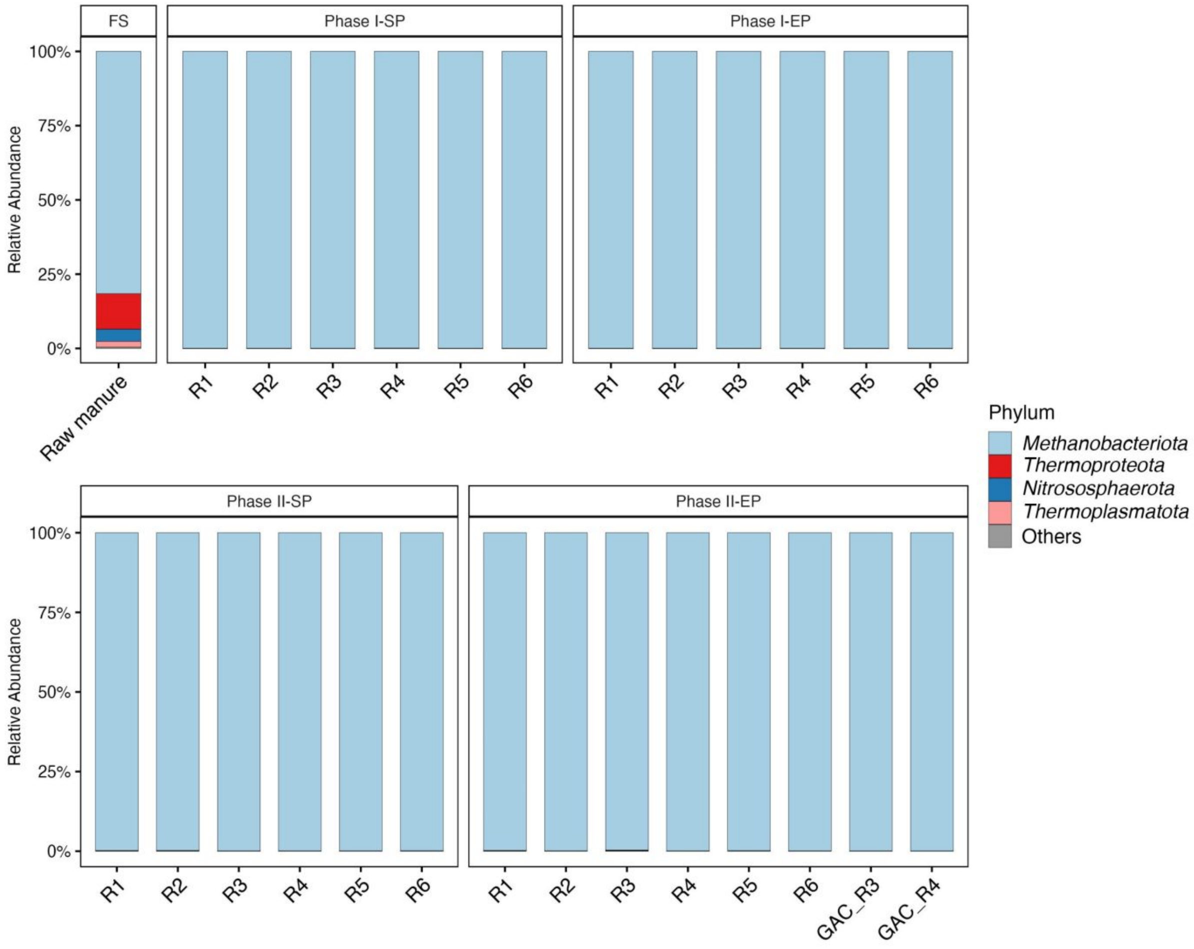

Figure S5. Archaeal community composition at the phylum level.

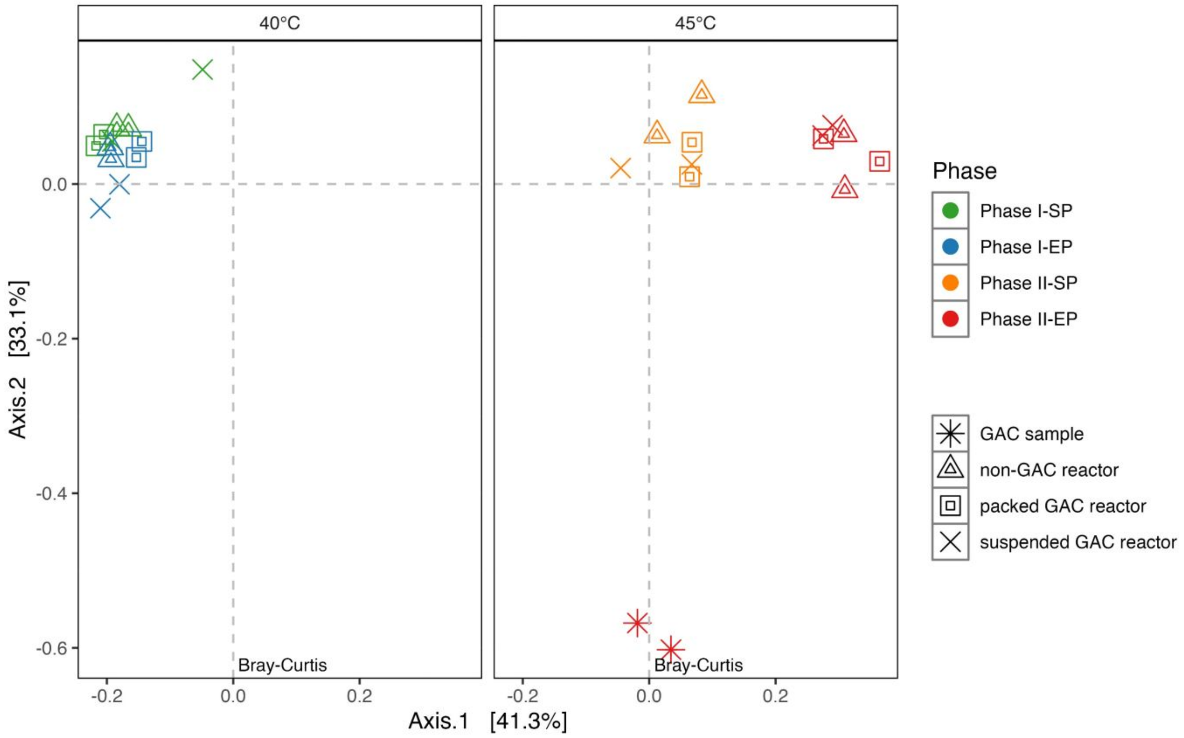

Figure S6. PCoA of the resistome using normalised abundance values.

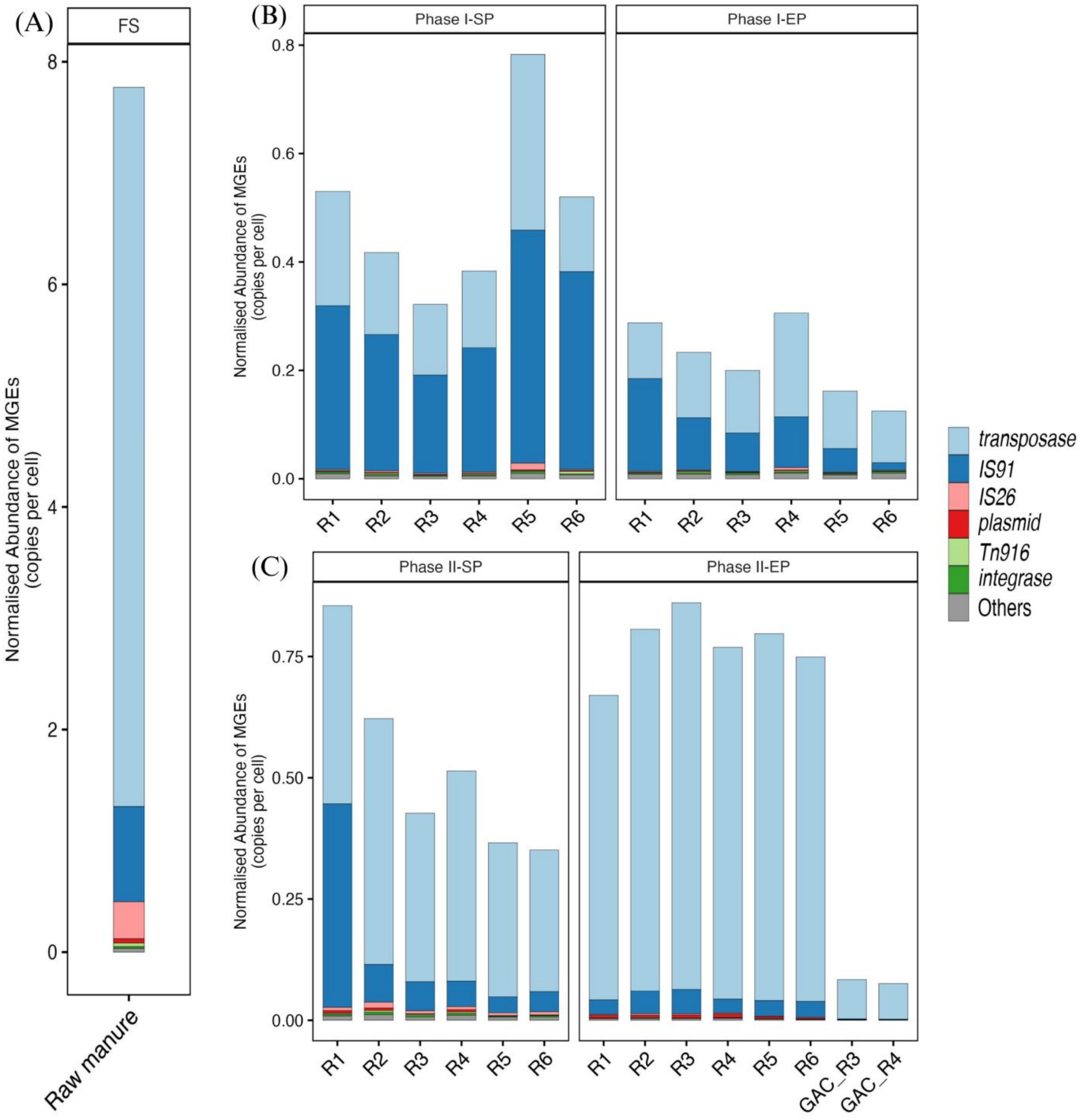

Figure S7. Normalised abundance of MGEs. (A) Raw manure. (B) Phase I. (C) Phase II. R1 & R2: non-GAC reactors; R3 & R4: packed GAC reactors; R5 & R6: suspended GAC reactors.

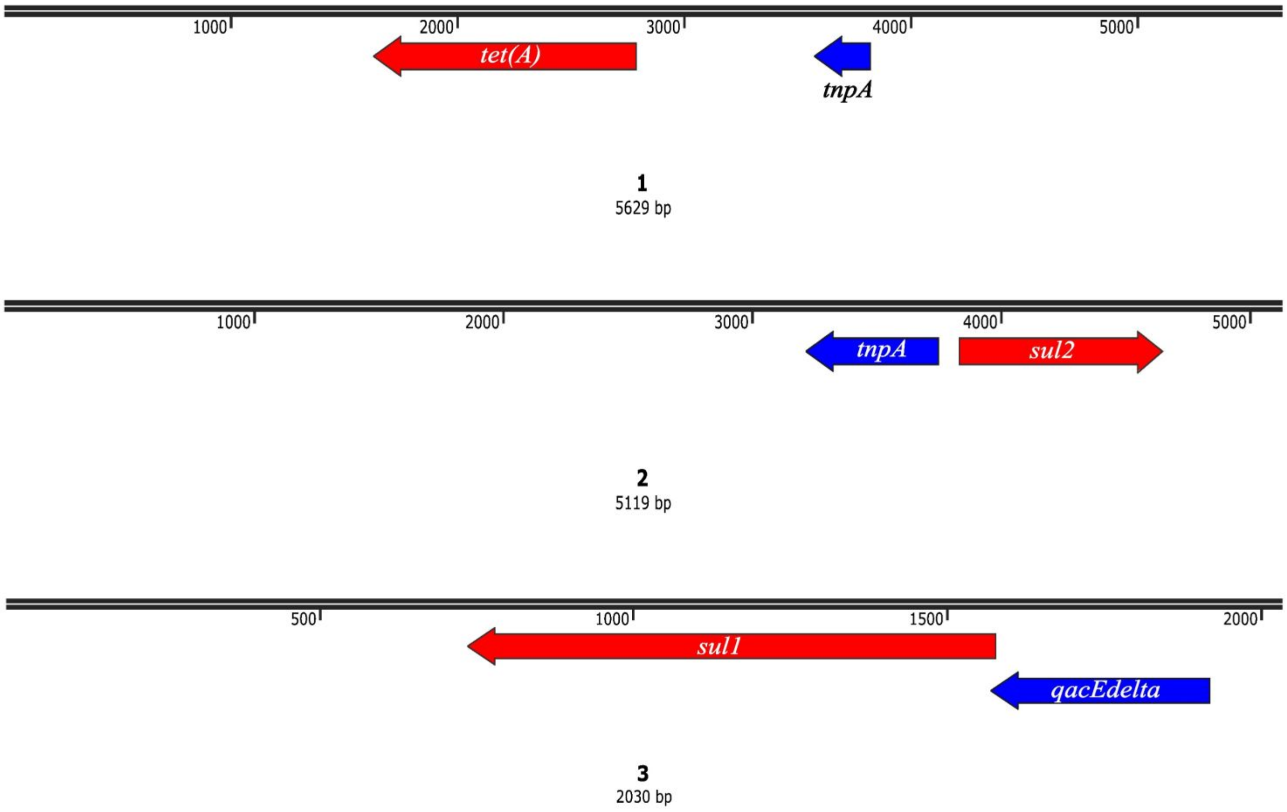

Figure S8. The co-occurrence pattern of ARGs and MGEs in contigs.
